# Influence of Nitrative Stress on the Synthesis of Neuromelanin Model Systems

**DOI:** 10.1021/acschemneuro.5c00676

**Published:** 2025-10-24

**Authors:** Silvia De Caro, Fabio Schifano, Enrico Monzani, Stefania Nicolis

**Affiliations:** † Department of Chemistry, 19001 University of Pavia, Via Taramelli 12, Pavia 27100, Italy; ‡ IUSS School for Advanced Studies of Pavia, Palazzo Del Broletto, Piazza della Vittoria 15, Pavia 27100, Italy

**Keywords:** dopamine, neuromelanin, Parkinson’s disease, spectroscopy and spectrometry, reactive nitrogen species, biomimetic models

## Abstract

Neuromelanin is a dark pigment present in the human brain and involved in the onset of Parkinson’s disease. Since natural pigment can only be extracted from the human brain in very small quantities, synthetic models of neuromelanin have been developed in recent years for research purposes, consisting of melanin conjugates and proteins made up of dopamine and β-lactoglobulin. Here, we studied the influence of nitrative stress on the synthesis of neuromelanin models, as it is known that reactive nitrogen species are present *in vivo* under pathophysiological conditions. HPLC-MS/MS analysis and ^1^H NMR and UV–vis absorption spectra show that nitration on the protein component does not affect the conjugate synthesis, while increasing nitration on the melanic component, by replacing dopamine with its nitrate derivative, 6-nitrodopamine, gradually inhibits the melanization. Moreover, although 6-nitrodopamine is not able to trigger melanization alone, in the presence of dopamine, it is incorporated into the conjugate. This study represents a step forward toward the synthesis of models that are increasingly similar to human neuromelanin, considering the influence of nitrative stress on neuromelanin pigment properties and biosynthesis.

## Graphical abstract

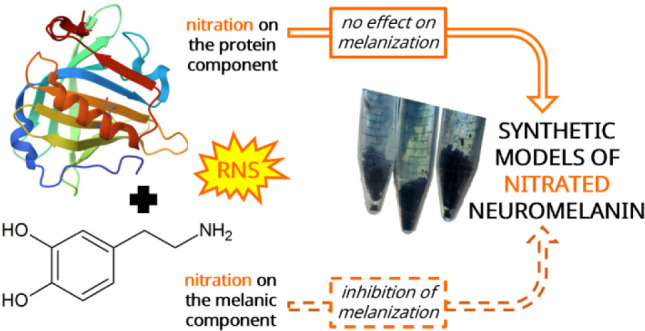

## Introduction

Parkinson’s disease (PD) is a neurodegenerative condition that is becoming more and more widespread in the last years; 6 million people worldwide were affected by this disease in 2016.^1,2^ To date, PD diagnosis is clinically based; the main symptoms related to this pathological condition are bradykinesia, rest tremor, balance loss, and rigidity, but nonmotor symptoms, like cognitive decline, depression, and pain, are involved as well.^1,3^ However, one of the most relevant characteristics of PD, as of other neurodegenerative diseases, is the slow progression of the disease; in fact, the disease itself could last ages, during which symptoms accumulate and get worse and worse.^1^ From a pathophysiological point of view, PD is characterized by α-synuclein (α-syn) aggregation, mitochondria, and lysosomal dysfunction, mainly related to dopamine (DA) oxidation, and neuroinflammation, which result in accelerated death of dopaminergic neurons, mainly in *substantia nigra* (SN) and *locus coeruleus* (LC) brain regions.^1,3,4^ Alterations in the iron, calcium, and glutamate pathways are also involved in the onset of PD.^3,5^

Another factor involved in PD pathophysiology is neuromelanin (NM), which is the subject of the present work. NM is a dark pigment found in dopaminergic neurons in SN and noradrenergic neurons in LC; in particular, it is contained in cytosolic organelles surrounded by a double membrane, suggesting an autophagic origin.^4–7^ Regarding the chemical structure, NM is formed by melanic, lipidic, and protein moieties, containing covalent links among the components.^5–7^ The melanic portion consists of a pheomelanin core surrounded by eumelanin aggregates on the surface, approximately in a 1:3 ratio, as confirmed by chemical degradation studies with H_2_O_2_, HI, and HCl. These species result from the polymerization of cysteinyl-DA and DA, respectively, by iron-mediated oxidation of DA to quinone species.^5,7,8^ The lipidic portion constitutes about 20% of the total mass of NM and is probably made up of dolichols and dolichoic acids;^9^ to date, NM has not yet been fully characterized due to its insolubility and complexity.^4,10^ Proteomic analysis of NM granules and organelles led to the identification of several hundred proteins.^11–13^ This protein portion is constituted by fibrillar aggregates with a β-cross structure; in fact, quinones derived from catecholamine (CA) oxidation are more prone to react with fibrils instead of native proteins.^7^ This structural feature of NM, confirmed by X-ray experiments, showing a 4.7 Å layer separation between the sheets, typical of fibril backbones, represents one of the differences between NMs and peripheral melanins. The latter are made of eumelanic oligomers packed in π-stacked layers without other components and exhibit the signature of π-stacked layers with a 3.5 Å separation. Peripheral melanins differ from NM for the biosynthesis as well; in fact, they are formed thanks to the enzymatic activity of tyrosinases and catechol oxidases.^4,8,10^ In addition to organic components, the NM structure also includes inorganic species, i.e., metal ions such as iron, copper, and zinc.^6^ Iron constitutes the main component of the metal portion of NM, exhibiting a concentration of 5–12 μg/mg.^5^ It is mainly present in multinuclear clusters of high-spin pseudo-octahedral centers, linked by oxo-hydroxo bridges, in a ferritin-like conformation; also, mononuclear centers bound to −OH groups or catechol’s O atoms have been detected.^5,7^

NM biosynthesis occurs in the cytosol of neurons as a consequence of an accumulation of CAs, mainly DA in SN and norepinephrine in LC, when their intracellular concentration exceeds 3 μM, due to an insufficient inclusion of the neurotransmitters in the synaptic vesicles.^7,8,14^ The biosynthesis starts with the oxidation of CAs to the corresponding quinones, which then react with nucleophilic residues such as the cysteine residues of peptides and proteins. The reaction proceeds with successive attachments of quinones, giving a lack of structural organization of the final species. During this process, iron ions promote catechol oxidation and remain trapped within the structure, interacting with the catechol’s oxo ligands.^7^ This iron-melanin-β-sheet complex cannot be degraded by the proteasome; so, it persists in the cytosol and reacts with dolichols and dolichoic acids to give NM.^4^ In human beings, NM biosynthesis starts early in life and NM accumulates over years, since no cellular excretion has been detected in physiological conditions and neurons do not possess any systems to degrade them.^10^

It has been reported that, in the human brain, NM possesses both neuroprotective and neurotoxic roles, depending on the cellular context.^15^ On the one hand, NM biosynthesis prevents the intracellular accumulation of DA, which can be neurotoxic because, when in excess, it is released by neurons and can react with reactive oxygen species (ROS) to give quinones, which in turn can lead to the formation of adducts with protein fibril seeds through the interaction with lysine, cysteine, and histidine residues, with consequent loss of function.^5,7,14^ Another reactive metabolite of DA is 3,4-dihydroxyphenylacetaldehyde (DOPAL), which mainly interacts with α-syn, forming a Schiff base with its lysine residues; this reaction is much faster than the interaction of α-syn with quinones, due to the lack of cysteine residues in the protein.^4^ Moreover, NM biosynthesis sequestrates toxins and iron ions, bringing them into an inactive state and preventing Fenton chemistry.^5,7,10^ On the other hand, after neuronal damage, NM can be released extracellularly and remains in the environment for a long time because of its insolubility and difficult degradation.^10^ It has been seen both in humans and in animal models that extracellular NM causes inflammation and microglia activation through phagocytosis, leading to the release of neurotoxic mediators, such as ROS. Consequently, dopaminergic neurons start to degenerate, creating a self-sustaining circle that leads to the rise of pathological conditions. In addition, extracellular NM can also release iron ions.^4,10^ It has also been established the relation between the vulnerability of neurons and their NM content.^10^

All these implications of NM in PD-related pathways underline the importance of further studies to better understand its role and, finally, to gain a clearer view of the overall pathophysiology of the disease. For example, NM is a good candidate to become a biomarker for PD through MRI.^14^ However, only a small amount of NM can be obtained from the human brain, 1.0 mg from SN and 0.1 mg from LC of at least 4 individuals.^14^ To this aim, over the past years, synthetic analogues of NM have been developed to elucidate the structure, properties, and reactivity of human NM. These analogues can, for example, be used to induce PD in animal models, to obtain conditions more similar to the development of the disease in the human brain.^7^ In particular, these analogues are made of DA and β-lactoglobulin (βLG), an 18.4 kDa lipocalin present in milk which contains three α-helices and a central β-barrel, called the calyx, with binding properties toward lipophilic molecules. βLG is easy to purify from milk and to convert into fLG, its fibrillated form, in which Cys_121_ is solvent-accessible and reactive, contrary to what is observed in the native protein, making it a good model for human neuronal proteins in the NM “core” and very convenient for producing samples in relatively large quantities for research purposes.^7,16^

Another pathological condition related to the onset of PD is the presence of oxidative and nitrative stress with increasing concentrations of ROS and reactive nitrogen species (RNS). RNS mainly consist of nitrogen monoxide (nitric oxide, NO), produced *in vivo* by nitric oxide synthase (NOS), and the products of its biochemical reactions. High levels of NO and NOS have been found in the brain of PD patients.^17^ NO has a protective role against inflammation, but upon NOS upregulation, it can lead to cell damage.^18^ NO can react with the superoxide radical anion during metabolism, forming the peroxynitrite anion (ONOO^–^), a species with a high nitrating power. RNS can react with macromolecules such as proteins, nucleic acids, and lipids, damaging them. In particular, an increased presence of 3-nitrotyrosine (3-NT) has been reported in several neurodegenerative disorders, and both 3-NT and 6-nitrodopamine (6-NDA), the nitrated derivative of DA, have been detected *in vivo* under nitrative stress conditions (Figure 1).^18–20^ Nitrogen dioxide, NO_2_ (deriving from NO aerobic oxidation), and the nitrite anion, NO_2_^–^, are mediators of the nitrating activity of NO, being less reactive than peroxynitrite but yet potentially toxic. NO_2_^–^ can nitrate phenols (for example, giving 3-NT) by acting as a substrate in the peroxidase activity of enzymes like myeloperoxidase.^18,20^

**Figure 1:**
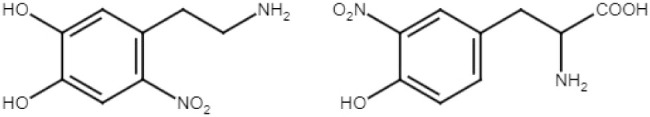
Structures of 6-NDA (left) and 3-NT (right).

Moreover, it has been reported that in a microglial cell culture treated with NM, an increase of the concentration of NO and NO_2_^–^ was detected.^21,22^ All this evidence together suggests that RNS and nitrative stress should be taken into consideration while studying the NM biosynthesis, since they may play a role in this process. On the other hand, the limited availability of human NM makes it difficult to identify nitrated proteins in the natural pigment, in which even innovative approaches have led to the identification of numerous proteins but did not allow to identify which protein is nitrated and to what extent,^11^ hence the importance of working on model systems.

The goal of this work is to investigate how the presence of nitrated species affects the synthesis of NM models and to obtain better models of human NM. To this aim, we synthesized nitrate derivatives of the reagents of the melanization process, βLG and DA, by treating them with RNS. In particular, we focused on the nitration of both the protein and the melanic portion of the NM, pretreating βLG with peroxynitrite and substituting DA with its nitrated derivative, 6-NDA (or using a mixture of the two species).

## Results and Discussion

In PD research, synthetic models of NM are used to help understand the characteristics and causes of disease development, in particular, by elucidating the mechanisms of NM biosynthesis and NM reactivity that could be related to the onset of pathological conditions. To date, the models used have been prepared without the presence of nitrate species. But, since nitration reactions and nitrated compounds have been observed in the brain, in particular, of PD subjects, in this work, we have developed the first synthetic models of nitrated NM.

To this end, we exploited the protocol developed by our group in the last years for the synthesis of NM models,^5,7^ using as reagents, in addition to βLG and DA, also their nitrated derivatives or mixtures of them. For all of the samples reported in Table 1 after air oxidation of the reagents, we obtained a brown solution with no presence of precipitate, suggesting that soluble melanin-protein conjugates were formed. In the case of (6-NDA)­EuβLG, the color of the solution was very light brown, suggesting that only little oligomerization occurred.

**Table 1:** NM Samples

EuβLG	DA (15 mg) + βLG (30 mg)
Eu(βLG-NO_2_)	DA (15 mg) + βLG-NO_2_ (30 mg)
(6-NDA)EuβLG	6-NDA (15 mg) + βLG (30 mg)
(DA/6-NDA)EuβLG	1:1 mixture of DA and 6-NDA (15 mg) + βLG (30 mg)

### Characterization of the NM Sample Nitrated on the Protein Portion: Eu­(βLG-NO_2_)

Two samples of NM were synthesized, one with the standard protocol, EuβLG, and the other one replacing the native protein with the prenitrated one, Eu­(βLG-NO_2_). To confirm that the nitrated protein was incorporated into the NM structure, after digesting the samples with trypsin and pepsin, an HPLC-MS analysis was performed, looking for the protein fragments containing nitrated residues. In particular, in the presence of RNS, nitration on Tyr (and much less probably on Trp) can occur, corresponding to the substitution of one H atom with a nitro group (mass increment of 45 Da).^23^ In the Eu­(βLG-NO_2_) sample, the fragment ^41^VYVEELKPTPEGDLEILLQK^60^ was found to be nitrated on the Y_42_ residue. In fact, both the chromatograms of the two samples show a peak with a retention time of 47 min, corresponding to the elution of the ^41^V–K^60^ fragment containing a native tyrosine residue in the 42 position, but only in the chromatogram of the Eu­(βLG-NO_2_) sample, a peak with a retention time of 48 min appears, corresponding to the elution of the same fragment, but containing a nitrated tyrosine residue in the 42 position, as confirmed by the MS spectra (Figure 2). This analysis confirms that a nitration reaction on the protein occurred.

**Figure 2:**
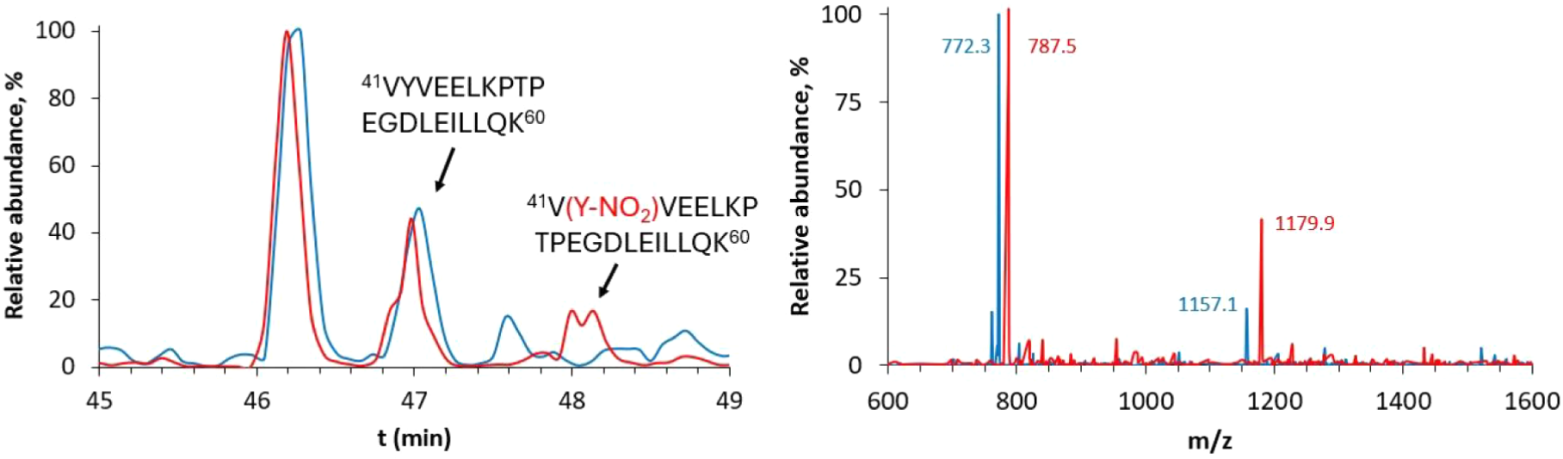
Left: HPLC-MS chromatograms (zoom from *t*_r_ = 45 min to *t*_r_ = 49 min) of EuβLG (blue) and Eu­(βLG-NO_2_) (red) injected in the HPLC-MS system after digestion. Right: MS spectra of the ^41^VYVEELKPTPEGDLEILLQK^60^ fragment (*m*/*z* 2312.3 (+), 1157.1 (2+), 772.3 (3+)) from EuβLG digestion (blue) and the ^41^V­(Y-NO_2_)­VEELKPTPEGDLEILLQK^60^ fragment (*m*/*z* 2357.3 (+), 1179.9 (2+), 787.5 (3+)) from Eu­(βLG-NO_2_) digestion (red).

We know from the literature that Y_99_ is another possible target of the nitration reaction on βLG, since it is solvent-exposed;^24,25^ however, we did not find any fragment containing Y_99_–NO_2_ in our analysis. The modified residue was probably present but could not be identified because it was likely hidden by the large number of DA quinones and related protein adducts present in the sample.

A further analysis was performed on the HPLC-MS data to investigate the modification occurred on the amino acid residues during the NM synthesis; in particular, we took into consideration the following types of modifications: (a) oxidation (i.e., insertion of an oxygen atom) of Cys and His residues: mass increment of 16 Da; (b) addition of DA or DA quinones on Cys, His, and Lys residues: mass increment of 151 or 149 Da, respectively.^26,27^

Our data indicate that C_121_ was oxidized and K_60_, K_69_, K_70_, K_75_, K_77_, and K_91_ were modified upon the addition of DA or DA quinones (data not shown).

As previously described for standard NM models, HPLC-MS analysis also allows the identification of the binding sites of the melanic portion on the protein since fragments containing the amino acid residues which bind the melanic oligomers are not detectable by this type of analysis. This is because either a species with a large and unknown molecular mass is bound to the amino acids or the binding of the melanic portion interferes with the interaction between the protein and the proteolytic enzymes, thus preventing the complete fragmentation.^7^ In particular, we focused our search on two types of amino acid residues, cysteine and histidine, since they possess side chains that are highly reactive toward DA quinones, which are involved in the melanization process. In the βLG sequence, there are two histidines (H_146_ and H_161_) and five cysteines (C_66_, C_106_, C_119_, C_121_, and C_160_) of which C_121_ is the only free (being not involved in disulfide bonds) and therefore potentially reactive.^16^ Among the three amino acids, H_146_, H_161_, and C_121_, which represent the possible binding sites of the melanic portion, H_146_ has previously been identified as the main binding site, since fragments containing it have been found in the analysis of the native protein but not in NM samples.^7^ This was confirmed also by this HPLC-MS analysis in the Eu­(βLG-NO_2_) sample: no fragments containing H_146_ were found. Regarding the other possible binding sites, H_161_ is close to the C-terminus of the protein chain, and sometimes, even in the native protein samples, peptides derived from the tryptic and peptic digestion close to the terminus of the protein sequence are too short to be detected by HPLC-MS analysis, so our results do not demonstrate whether or not H_146_ is a binding site. Whereas, C_121_ does not react with DA quinones because, as mentioned above, it undergoes oxidation before having the possibility to react with other species.

To understand the effect of nitration on Y_42_ on the melanization process, we recorded the ^1^H NMR spectra of the two samples EuβLG and Eu­(βLG-NO_2_) (and, for comparison purposes, the native βLG, Figure 3).

**Figure 3:**
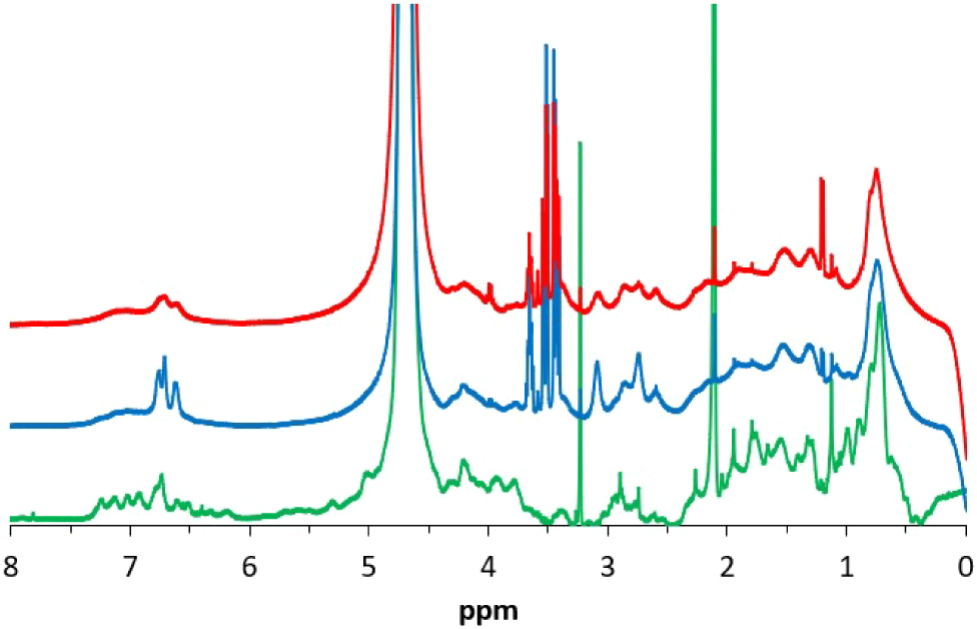
^1^H NMR spectra of native βLG (green), EuβLG (blue), and Eu­(βLG-NO_2_) (red).

The interpretation of the effect of melanization on the NMR signals is not trivial, as the observed effects following protein modification can be discordant. The hydrogen atoms of the protein amino acid residues show sharp peaks when they have mobility in solution; this occurs with small (fast-rotating) molecules or unstructured (and mobile) residues of larger molecules in solution.

During the βLG (or βLG-NO_2_) melanization with DA, if DA polymerization is faster than the covalent linking with the protein, βLG binds to large DA oligomers, resulting in very large (and slow-rotating) molecules. At the opposite, if the covalent linking is fast, βLG undergoes modification with much smaller DA oligomers, thus inducing a loss in structure and an increased local mobility.

Figure 3 shows that, upon melanization, the amide proton signals (6.5–7.5 ppm), visible in βLG, disappear; probably, due to a partial denaturation of the protein near the catechol binding sites, an easier exchange with the deuterated solvent occurs with EuβLG and Eu­(βLG-NO_2_). At the same time, the latter conjugates show a broad signal in the same region, associated with the aromatic residues of the melanic component. Broadening of the signals indicates that the proton T_2_ relaxation time has been shortened in the conjugates due to the reduced conformational mobility of the protein chain together with the slower rotational motion in solution, both due to the melanization reaction.

Moreover, in EuβLG and Eu­(βLG-NO_2_) spectra, a broad singlet around 3.3 ppm appeared, a typical signal reported in the literature for similar conjugates.^5^ So, we can conclude that nitration of the protein portion does not significantly affect the melanization process.

### Characterization of the NM Samples Nitrated on the Melanic Portion: (DA/6-NDA)­EuβLG and (6-NDA)­EuβLG

Besides having assessed that the nitration of the protein portion does not affect the melanization reaction, we investigated the effect of the nitration on the melanic portion. To this aim, we synthesized two NM samples and compared them with those of the previous EuβLG: (6-NDA)­EuβLG, replacing DA with its nitrated derivative, 6-NDA, and (DA/6-NDA)­EuβLG, using a 1:1 mixture of DA and 6-NDA in the melanization process.

The UV–vis spectra of the three samples just after mixing the reagents (*t* = 0) and after 4 days at the end of the synthesis are shown in Figure 4.

**Figure 4:**
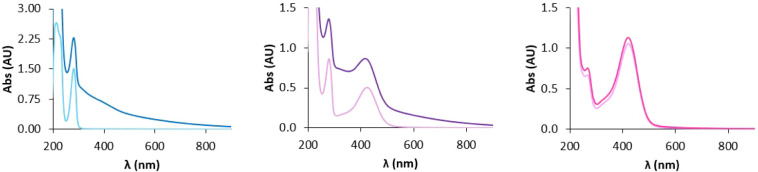
UV–vis spectra of EuβLG (blue), (DA/6-NDA)­EuβLG (violet), and (6-NDA)­EuβLG (pink) at *t* = 0 (light) and after 4 days (dark).

We can notice that in addition to the band around 280 nm, related to DA, the spectra of (6-NDA)­EuβLG and (DA/6-NDA)­EuβLG also show a band at 420 nm, related to 6-NDA. The melanization process can be highlighted by two main changes in the UV–vis spectrum: (a) an increase in the background absorption, mainly at low wavelength values, due to the light scattering promoted by the NM and (b) a decrease in the DA absorption band, indicating that its concentration in solution decreases due to its incorporation into the NM.

These changes are present, not only in the spectrum of EuβLG but also in that of (DA/6-NDA)­EuβLG, even if with a smaller intensity; on the other hand, they are almost absent in the spectra of (6-NDA)­EuβLG. These data indicate that 6-NDA alone is not able to lead to the formation of a NM polymer and only promotes the formation of small adducts that do not cause light scattering.

To clarify this point, the ^1^H NMR spectra of the three samples were registered and compared with the one of native βLG, as previously done for the characterization of the NM sample nitrated on the protein portion (Figure 5).

**Figure 5:**
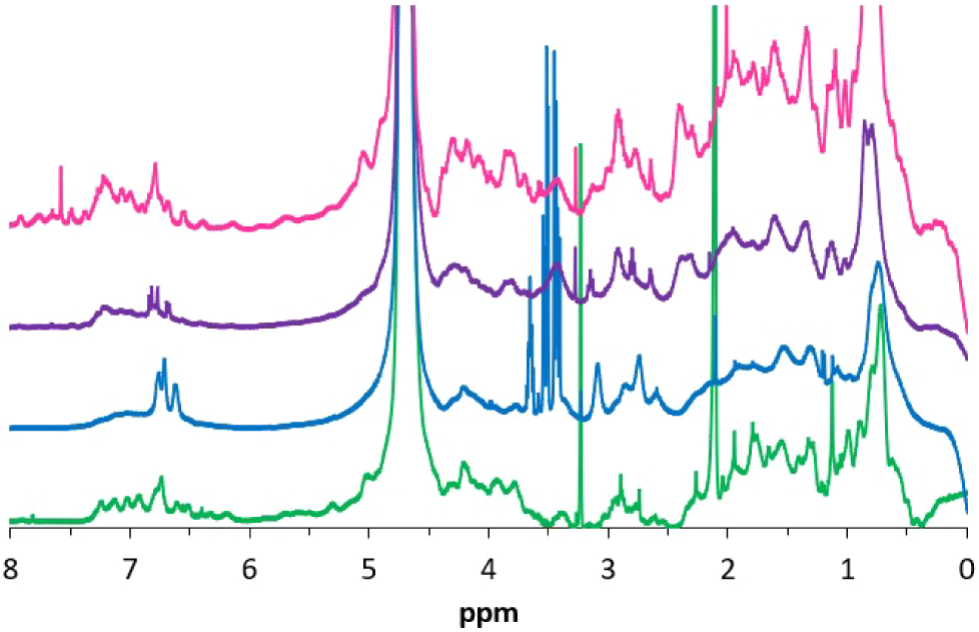
^1^H NMR spectra of native βLG (green), EuβLG (blue), (DA/6-NDA)­EuβLG (violet), and (6-NDA)­EuβLG (pink).

The spectra of EuβLG and (DA/6-NDA)­EuβLG show the typical features of an NM sample, as described by the broadening of the signals and the appearance of a broad singlet at ≈3.3 ppm. In contrast, in the spectrum of (6-NDA)­EuβLG, there are more peaks, the signals appear sharper, and the singlet at 3.3 ppm is much less pronounced, indicating either that a lower fraction of melanic oligomers is bound to the protein or that the oligomers themselves are smaller. Both situations result from a lower yield of the melanization reaction when only 6-NDA is present as a catechol during the synthesis.

To investigate the melanization degree of the three samples, HPLC-MS analysis was performed. The fragment ^142^ALPMHIR^148^, containing H_146_ (i.e., the main binding site), was used as a marker of the melanization degree of the samples, as explained before. From the chromatograms of the three samples shown in Figure 6, it can be seen that the intensity of the peak with a retention time of 27.6 min, corresponding to the elution of the ^142^A-R^148^ fragment, increases upon DA nitration grade, being low in EuβLG, higher in (DA/6-NDA)­EuβLG, and much higher in (6-NDA)­EuβLG. This experiment suggests that nitration on the catechol hinders the melanization process, without quenching it completely: melanization occurs even in the (6-NDA)­EuβLG sample but with a much smaller yield.

**Figure 6:**
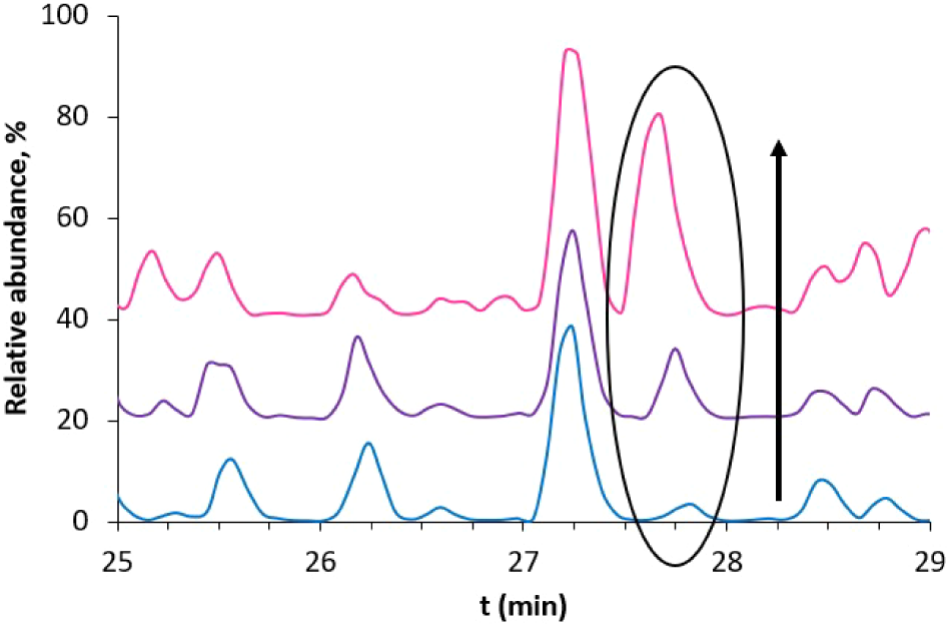
HPLC-MS chromatograms (zoom from *t*_r_ = 25 min to *t*_r_ = 29 min) of EuβLG (blue), (DA/6-NDA)­EuβLG (violet), and (6-NDA)­EuβLG (pink) injected in the HPLC-MS system after digestion.

The HPLC-MS, UV–vis, and ^1^H NMR data confirm that 6-NDA alone is not able to give an extended melanization process. Most probably, the nitro group on the aromatic residue in 6-NDA increases the one-electron redox potential of the catechol with respect to that of DA (*E* ^0^ = 0.752 V^28^), making 6-NDA less prone to be oxidized and, consequently, slowing down the melanization process.

Once assessed that 6-NDA is not able to lead the melanization reaction when being the only catechol component, it remains to clarify its destiny when both DA and 6-NDA are present in the reaction mixture. To this aim, we performed an HPLC analysis on the (DA/6-NDA)­EuβLG sample both at the beginning (*t* = 0) and at the end (*t* = 4 days) of the melanization process (Figure 7).

**Figure 7:**
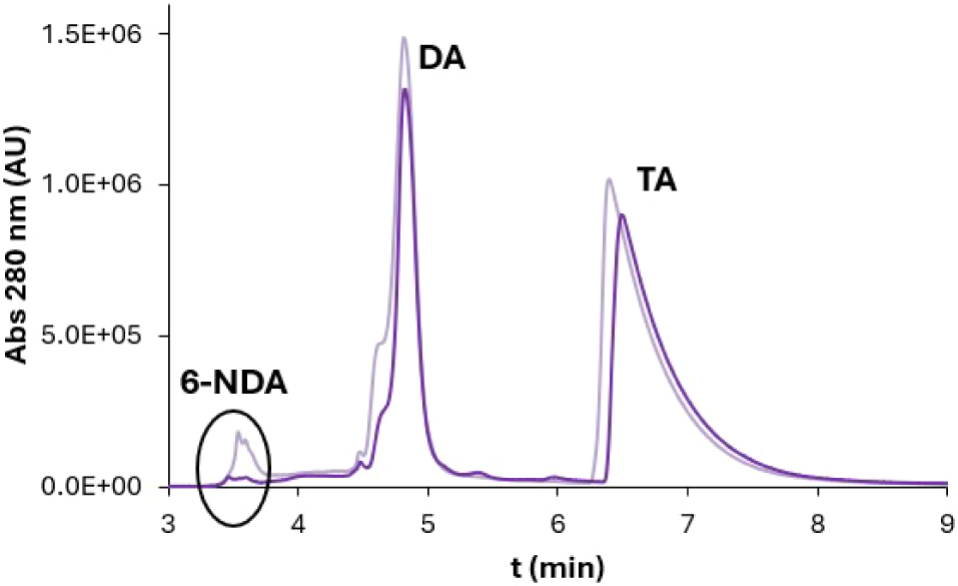
HPLC chromatogram (zoom from *t*_r_ = 3 min to *t*_r_ = 9 min) of (DA/6-NDA)­EuβLG at *t* = 0 (light) and after 4 days (dark). The three signals correspond to 6-NDA (*t*_r_ = 3.5 min), DA (*t*_r_ = 4.8 min), and the internal standard tryptamine (TA) (*t*_r_ = 6.4 min).

This analysis shows that the signal related to 6-NDA is clearly detectable before the reaction, and its intensity is much lower after the melanization process occurred. This decrease is visible even when comparing the areas of the DA and 6-NDA signals, normalized by dividing each of them by the area of the TA signal (Table 2): the 6-NDA/DA ratio decreases from 0.09 for the sample at *t* = 0 days to 0.02 for the sample at *t* = 4 days. These data prove that 6-NDA has disappeared from the solution and, therefore, has been incorporated into the melanic pigment.

**Table 2:** Areas of the Signals in the Chromatograms in Figure 7 and Their Ratios

*t* (days)	6-NDA	DA	TA	DA/TA	6-NDA/TA	6-NDA/DA
0	2.0 × 10^6^	2.4 × 10^7^	3.0 × 10^7^	0.78	0.07	0.09
4	4.2 × 10^5^	1.8 × 10^7^	2.8 × 10^7^	0.63	0.02	0.02

## Conclusions

To better elucidate the neurochemical frame in which PD starts to develop and to gain information to synthesize models that better mimic the human NM, we considered how the presence of RNS can affect NM biosynthesis. As results from our study, nitration on the protein portion of our NM model does not have a relevant influence on its synthesis; in fact, the ^1^H NMR spectra of the EuβLG and Eu­(βLG-NO_2_) samples exhibit no significant differences. On the contrary, nitration on the melanic portion has both the effects of making oligomerization more difficult and, because of the slower process, producing smaller adducts. Indeed, it has been seen that by completely replacing DA with 6-NDA, the melanization is inhibited, and by replacing DA with a 1:1 mixture of DA and 6-NDA, the NM forms but with a lower yield. This conclusion comes from the evidence of UV–vis, ^1^H NMR, and HPLC-MS/MS characterization of (6-NDA)­EuβLG and (DA/6-NDA)­EuβLG, in comparison with EuβLG. First, in the UV–vis spectra, the increase of the background absorption and the decrease of the DA band are reduced in the sample of NM synthesized with the mixture of catechols and completely absent in the sample with only the nitrated catechol; then, in the ^1^H NMR spectrum of the (6-NDA)­EuβLG sample, the typical features of a NM spectrum are absent; finally, the HPLC-MS/MS analysis indicates that the concentration of the fragment which constitutes the binding site of the melanic portion to the protein backbone increases together with the nitration grade of DA, indicating a lower and lower melanization yield. Moreover, thanks to an HPLC analysis, we assessed that in the presence of a 1:1 mixture of DA and 6-NDA, melanization occurs due to the presence of DA and the nitrated species is incorporated in the melanic polymer as well.

With this work, through the characterization of synthetic NM samples with nitrative modification on either the melanic or the protein portion, we provided an early overview of the effect of the nitrative stress on NM synthesis. In the future, an interesting development could be the synthesis of NM models in which coexist the melanic and the protein components nitrated, in addition to metal ions (iron and copper) and the study of the reactivity of these NM samples. Even if these are *in vitro* studies, they point toward the direction of making synthetic NM samples more and more similar to the pigment present *in vivo*, which could be used in cellular models.

## Methods

### Instruments

All chromatographic separations were performed with a Shimadzu Prominence instrument equipped with a DGU-20A3R degassing unit, two LC-20AD pumps, a SPD-M20A photodiode array (190–800 nm wavelength range), and a CTO-20A column oven. A Supelco Supelcosil LC-18 (5 μm, 250 × 10 mm) semipreparative column was used as the solid phase. UV–vis spectra were recorded on an Agilent 8453 spectrophotometer equipped with a 1024 photodiode array detector (190–1100 nm wavelength range) and a quartz optical cell of 1 cm path length. Mass spectrometry analyses were performed on a Thermo Finnigan LCQ ADV MAX ion-trap mass spectrometer with an ESI ion source. The ESI conditions were as follows: capillary temperature of 210 °C, tube lens voltage of −25 V, and source voltage of +4.9 kV. The system was run in automated LC-MS/MS mode, using a Surveyor HPLC system equipped with a Phenomenex Jupiter 4U Proteo column (4 μm, 150 × 2.0 mm). For the analysis of protein fragments, Bioworks 3.1 and Xcalibur 2.0.7 SP1 software were used. ^1^H NMR spectra were recorded on a Bruker AVANCE 400 spectrometer, operating at 9.37 T and 400 MHz, and analyzed with the software TopSpin 1.3.

### Materials and Stock Solutions

All reagents were purchased from Merck at the highest available purity and were not further purified. Phosphate buffer, 50 mM at pH = 7.0, was prepared by dissolving the appropriate amounts of NaH_2_PO_4_ and Na_2_HPO_4_ solid salts in Milli-Q water. The pH was adjusted by adding droplets of an aqueous concentrated NaOH solution. DA stock solutions were prepared in 10 mM HCl. βLG stock solutions were prepared in Milli-Q water. Peroxynitrite was synthesized as previously reported.^29^ 6-NDA was synthesized by nitration of DA: a solution of 25 mg of DA in 10 mL of Milli-Q water was added with 5 equiv of NaNO_2_ and a drop of concentrated H_2_SO_4_ and left to react for 10 min in an ultrasound bath. 6-NDA was then purified by HPLC (in isocratic conditions with only Milli-Q water containing 0.1% of trifluoroacetic acid (TFA) and with a flow rate of 4 mL/min), lyophilized, resuspended with water and HCl, and lyophilized again; 6-NDA was obtained and stored as a hydrochloride salt. TA stock solution was prepared by dissolving 7.3 mg of TA in ethanol.

### NM Synthesis

In a plastic tube, light-shielded with tinfoil, 15 mg of DA and 30 mg of βLG were dissolved in 6 mL of 50 mM phosphate buffer at pH 7.0 and allowed to react in air at 37 °C for 4 days. Then, the conjugates were dialyzed with a 10 kDa cutoff against Milli-Q water for 3 days, replacing water at least 6 times, in order to remove unreacted DA and smaller DA-quinone oligomers. The conjugates were then lyophilized. This protocol was adapted for samples with prenitrated reagents, as reported in Table 1. In particular, 16 equiv of peroxynitrite were used to perform the βLG nitration and obtain βLG-NO_2_, since an excess (4 equiv) is needed for each tyrosine residue of the protein, and βLG contains four tyrosines in its structure. Pretreatment was performed in 50 mM phosphate buffer at pH 7.0, the same solvent used for the melanization process, because the peroxynitrite stock solution is strongly basic, due to stability reasons of the anion. The reaction was carried out at room temperature, and the nitration reaction was almost instantaneous: only a few seconds after adding the peroxynitrite to the βLG solution and mixing, the solution can be mixed with DA to start melanization. No dialysis is needed since the eventually unreacted peroxynitrite degrades to nitrate in a very short time at pH 7.

### HPLC-MS/MS Characterization

For the HPLC-MS analysis, 1 mg of each NM sample was dissolved in 1 mL of 20 mM ammonium bicarbonate buffer, pH 8.0. To denature the protein portion of NMs and break disulfide bonds, 8 M urea and 50 mM dithiothreitol (final concentration) were added, and the samples were left in an oil bath at 60 °C for 1 h; then, to prevent disulfide bond formation, 200 mM iodoacetamide was added, and the samples were incubated at 37 °C for 1 h. Unreacted reagents were removed by dialyzing the samples overnight with ammonium bicarbonate buffer. Protein digestion was performed initially by adding to each sample an aqueous solution of trypsin (1 mg/mL) in a 1:50 (w/w) ratio and incubating at 37 °C for 24 h and subsequently after acidification with one drop of concentrated HCl by adding an aqueous solution of pepsin (1 mg/mL) in a 1:50 (w/w) ratio and incubating at 37 °C for 24 h. Elutions were carried out with Milli-Q water added with 0.1% formic acid as solvent A and acetonitrile added with 0.1% formic acid as solvent B, with a flow rate of 0.2 mL/min. The solvent gradient started with 98% solvent A for 5 min, followed by a linear gradient from 98% to 55% solvent A in 65 min.

### ^1^H NMR Characterization

For the ^1^H NMR analysis, 1 mg of each NM sample was dissolved in 1 mL of D_2_O and incubated under stirring for 24 h before recording the spectrum. To overcome the low sample concentration, a large number of scans (up to 2400) were acquired.

### HPLC Analysis for 6-NDA Quantification

For 6-NDA quantification, 100 μL (the analysis has been carried out before lyophilization) of the (DA/6-NDA)­EuβLG sample in a total volume of 2 mL of Milli-Q water were injected in HPLC, both at the beginning (*t* = 0) and at the end (*t* = 4 days) of the melanization process. Before injecting, 10 μL of TA (stock solution 46 mM) as an internal standard were added to each sample. The elution was carried out in isocratic conditions, with only Milli-Q water containing 0.1% TFA, at a flow rate of 4 mL/min. The UV–vis absorption signal of the detector was followed at 280 nm (the wavelength at which both the analytes DA and 6-NDA and the internal standard TA strongly absorb).

## Abbreviations

3-NT: 3-nitrotyrosine
6-NDA: 6-nitrodopamine
α-syn: α-synuclein
βLG: β-lactoglobulin
CA: catecholamine
DA: dopamine
DOPAL: 3,4-dihydroxyphenylacetaldehyde
LC: *locus coeruleus*
NM: neuromelanin
NOS: nitric oxide synthase
PD: Parkinson’s disease
RNS: reactive nitrogen species
ROS: reactive oxygen species
SN: *substantia nigra*
TA: tryptamine
TFA: trifluoroacetic acid
